# Correlation of nasopharyngeal cultures prior to and at onset of acute otitis media with middle ear fluid cultures

**DOI:** 10.1186/s12879-014-0640-y

**Published:** 2014-12-05

**Authors:** Ravinder Kaur, Katerina Czup, Janet R Casey, Michael E Pichichero

**Affiliations:** Center for Infectious Diseases and Immunology, Rochester General Hospital Research Institute, 1425 Portland Avenue, Rochester, 14621 NY USA; Otitis Media Research Center, Legacy Pediatrics, Rochester, NY USA

**Keywords:** Acute otitis media, Tympanocentesis, Streptococcus pneumoniae, Haemophilus influenzae (non-typeable), Moraxella catarrhalis

## Abstract

**Background:**

We sought to determine if nasopharyngeal (NP) cultures taken at times of healthy visits or at onset of acute otitis media (AOM) could predict the otopathogen mix and antibiotic-susceptibility of middle ear isolates as determined by middle ear fluid (MEF) cultures obtained by tympanocentesis.

**Methods:**

During a 7-year-prospective study of 619 children from Jun 2006-Aug 2013, NP cultures were obtained from 6-30 month olds at healthy visits and NP and MEF (by tympanocentesis) at onset of AOM episodes.

**Results:**

2601 NP and 530 MEF samples were collected. During healthy visits, *S. pneumoniae* (*Spn*) was isolated from 656 (31.7%) NP cultures compared to 253 (12.2%) for Nontypeable *Haemophilus influenzae* (NT*Hi*) and 723 (34.9%) for *Moraxella catarrhalis* (*Mcat*). At onset of AOM 256 (48.3%) of 530 NP samples were culture positive for *Spn*, 223 (42%) for NT*Hi* and 251 (47.4%) for *Mcat*, alone or in combinations. At 530 AOM visits, *Spn* was isolated from 152 (28.7%) of MEF compared to 196 (37.0%) for NT*Hi* and 104 (19.6%) for *Mcat*. NP cultures collected at onset of AOM but not when children were healthy had predictive value for epidemiologic antibiotic susceptibility pattern assessments.

**Conclusions:**

NP cultures at onset of AOM more closely correlate with otopathogen mix than NP cultures at healthy visits using MEF culture as the gold standard, but the correlation was too low to allow NP cultures to be recommended as a substitute for MEF culture. For epidemiology purposes, antibiotic susceptibility of MEF isolates can be predicted by NP culture results when samples are collected at onset of AOM.

**Electronic supplementary material:**

The online version of this article (doi:10.1186/s12879-014-0640-y) contains supplementary material, which is available to authorized users.

## Background

Acute otitis media (AOM) is one of the most common diseases in childhood and causes a considerable illness burden for children. *Streptococcus pneumoniae* (*Spn*), Nontypeable *Haemophilus influenzae* (NT*Hi*) and *Moraxella catarrhalis* (*Mcat*) are the 3 main pathogens causing AOM [1],[2]. The “gold-standard” for etiologic diagnosis of AOM is by detecting pathogens in middle ear fluid (MEF) by culture [1],[2]. However tympanocentesis is not routinely performed to obtain and culture MEF, so scientists and clinicians are in a conundrum regarding scant data to allow recommendations for treatment of AOM or sinusitis, leading to the suggestion that there may be no choice but to rely on nasopharyngeal cultures (NP) [3].

NP cultures have been used in the past as an epidemiologic tool to monitor the mix of otopathogens circulating in a country or region, and their antibiotic susceptibility pattern [4]. However, in a recent systematic review van Dongen *et al* [5] found that concordances between NP samples collected at onset of AOM compared to MEF isolates vary from 68% to 97% per microorganism. For the most prevalent microbes, positive predictive values were about 50%. Most negative predictive values were moderate to high, with a range from 68% up to 97%. The results indicate that NP samples do not provide an accurate proxy for those of middle ear fluid.

In 2006, we began a longitudinal, multi-year, prospective study of AOM with a primary objective to further understand the immunologic mechanisms responsible for the otitis prone children. During the course of our studies, we collected NP samples and MEF from these children. We have periodically reported one microbiologic aspects of our research [6]-[15]. Here we sought to answer several questions regarding the value of NP cultures as to their concordance with MEF cultures collected by tympanocentesis. Can NP samples taken during healthy visits serve as a surrogate for MEF cultures? In the current pneumococcal conjugate vaccine era, where otopathogen mix has changed due to vaccine efficacy, are NP cultures taken at onset of AOM concordant with MEF cultures? Can antibiotic susceptibility of otopathogens collected from the MEF be predicted by otopathogen isolates obtained from the NP at healthy or at onset of AOM visits?

## Methods

### Study population

The details of the study design have been previously described [16]. Study participants were recruited mainly from a single private pediatric office (Legacy Pediatrics) in Rochester, NY. Four other private pediatric groups joined in the recruitment effort by referral of patients to Legacy Pediatrics. Written informed parental consent was obtained prior to any study procedures. This study was approved by the University of Rochester IRB and subsequently by the Rochester General Hospital IRB.

### Eligibility

Children were enrolled into the study at the age of 6 months and prospectively followed until 30-36 months of age (recently extended to 60 months of age). Inclusion criteria were: healthy, full term birth, no craniofacial anomalies and no known immune deficits. Participants were required to receive all doses of Pneumococcal Conjugate Vaccine (PCV) according to the U.S. schedule; either PCV7 or PCV13 depending on the date of their enrollment. This is an ongoing prospective study where not all the children have completed the planned visits. In addition, although we request collecting samples at 6, 9, 12, 15, 18, 24 and at 30-36 months of age, most parents don’t consent for all the seven collection visits, especially since a venipuncture occurs simultaneously with the NP samplings. There is no statistically definable pattern of missing data [16].

### Sampling

Nasal wash (NW) and nasopharyngeal swab (NP) samples were collected over 7 years (June 2006 to August 2013), prospectively from healthy children at 6, 9, 12, 15, 18, 24 and 30-36 months of age. During visits with AOM, MEF was obtained and cultured (both or a single tympanocentesis procedure depending on whether the infection was bilateral or unilateral) along with NW and NP swabs. Diagnosis of AOM was performed by validated otoscopists when children with acute onset of symptoms consistent with AOM had tympanic membranes (TMs) that were: (1) bulging or full, and (2) a cloudy or purulent effusion was observed, or the TM was completely opacified, and (3) TM mobility was reduced or absent. Sampling procedures, microbiology processing and identification, and molecular testing for organism identification have been previously described [6],[8],[15]. The oxacillin sensitivity of *S. pneumoniae* isolates was determined using Taxo™ P Discs (Beckton, Dickinson). Most *Spn* were also tested for their penicillin antibiotic susceptibility along with other antibiotics using VITEK 2 Gram Positive Susceptibility Card-AST-GP68 (BioMerieux, Inc) with the VITEK2 systems as described previously [17]. Microbiology data gathered from NW and NP are collectively represented as NP culture results. The detection rate of otopathogens from NW is higher than NP samples as shown recently by our group [18].

### Statistical analysis

All statistical analyses were conducted using GraphPad Prism. Positive cultures for *Spn*, NT*Hi* or *Mcat* from a MEF samples were defined as the gold standard for the etiologic diagnosis of AOM. In this analysis, 2 MEF samples obtained at the same visit were regarded as one case of AOM and any otopathogen found in either or both of these samples was treated as a single finding in that case. The NW and NP swab results (hereafter referred to as NP cultures) and MEF culture results were compared with χ^2^ test. The positive predictive value (PPV) represents the proportion of NP samples that tested positive for a NT*Hi* or *Spn* or *Mcat*, for which the paired MEF sample was also positive. The negative predictive value (NPV) represents the proportion of NP samples that tested negative for a common otopathogens, for which the paired MEF sample was also negative. The sensitivity represents the proportion of MEF samples that tested positive for NT*Hi* or *Spn* or *Mcat*, for which the paired NP sample was also positive. The specificity represents the proportion of MEF samples that tested negative for NT*Hi* or *Spn* or *Mcat*, for which the paired NP sample was also negative. Bacterial otopathogens between healthy vs AOM visits were compared using logistic regression with bacterial presence as binary outcome and visit type factor variable as predictor. A subject level random effect was included to model within-subject correlation. The function glmer() from the R package lme4 was used to calculate the model [19]. Estimates of the bacterial otopathogen presence rate and the visit group odds ratio were calculated directly from the model. The effects of age on the pathogens distribution during healthy colonization and AOM were assessed by Pearson correlation.

## Results

### Study population

During the 7-years involved in the current report 619 children were enrolled in the study. There were 2071 healthy visits among the children. The distribution of sample visits at 6, 9, 12, 15, 18, 24 and 30-36 months was as follows: 402 (19.4%), 388 (18.7%), 366 (17.7%), 297 (14.3%), 292 (14.1%), 244 (11.8%) and 82 (4%) respectively. A total of 530 AOM visits occurred in 309 children. The mean age of children at the time of AOM episode was 13.7 months and median age was 12.

### Comparison of otopathogen mix during NP colonization at healthy and AOM visits

During healthy visits, *Spn* was isolated from 656 (31.7%) NP cultures compared to 253 (12.2%) for NT*Hi* and 723 (34.9%) for *Mcat* alone or in combination (Table 1). At the onset of AOM, 256 (48.3%) of 530 NP samples were culture positive for *Spn*, 223 (42%) for NT*Hi* and 251 (47.4%) for *Mcat*, alone or in combinations (Table 1). There was a significant difference (p < 0.0001) between healthy and at onset of AOM NP cultures. Data show that NT*Hi* is more prevalent at AOM visits (Odd Ratio = 2.72). Co-colonization with multiple otopathogens distribution among healthy vs AOM visits are also shown in Table 1. The ratio of frequency of NT*Hi* (AOM/healthy) is about 200% and this estimate does not depend on the co-pathogens. On the other hand, the ratio of frequency (AOM/healthy) of *Mcat* alone or *Spn* alone are about 50-60% (ie. less prevalent in AOM visits). For *Spn*-*Mcat* [no NT*Hi*] the ratio of frequency (AOM/healthy) is about 100%, ie. equally prevalent. When *Mcat* or *Spn* is co-colonized with NT*Hi*, there is an NT*Hi* ratio of frequency of about 200% indicating NT*Hi* seems to dominate. Otopathogens patterns change during the transition to AOM. For example, a *Mcat*-alone colonization is more likely to co-colonize during the transition to AOM than NT*Hi*-alone colonization would be. This would explain the under-representation of *Mcat*-alone colonizations among AOM visits, without having to assume that *Mcat*-alone has a smaller transition rate to AOM. Co-colonization with multiple otopathogens was significantly higher (p < 0.0001) during AOM compared to healthy visits (Table 1). *Staphylococcus aureus* was also detected in 189 (9.1%) cases during healthy visits and 34 (6.4%) cases during AOM visits.

**Table 1:** **Comparison of NP Colonization at Healthy and AOM Visits along with MEF isolates during AOM**

Isolates	Healthy NP colonization	AOM NP colonization	AOM MEF
	Number (%)	Number (%)	Number (%)
*S. pneumoniae*	292 (14.1)	70 (13.2)	110 (20.8)
Nontypeable *H. influenzae*	97 (4.7)	97 (18.2)	166 (31.3)
*M. catarrhalis*	382 (18.4)	78 (14.7)	71 (13.4)
NT*Hi* + *S. pneumoniae*	60 (2.9)	52 (9.8)	19 (3.6)
*M. catarrhalis* + *S. pneumoniae*	245 (11.8)	99 (18.6)	22 (4.2)
NT*Hi* + *M. catarrhalis*	37 (1.8)	39 (7.3)	10 (1.9)
NT*Hi* + *M. catarrhalis* + *S. pneumoniae*	59 (2.8)	35 (6.6)	1 (0.2)
Other bacteriaǂ	870 (41.9)	35 (6.6)	44 (8.3)
No Bacteria	36 (1.7)	27 (5.1)	87 (16.4)

ǂOther bacteria were only sought when canonical otopathogens (*S. pneumoniae*, NT*Hi* and *M. catarrhalis*) were not detected in the visits. They may have been present along with main otopathogens but were not included in the calculations.

### Correlation of MEF otopathogens during AOM with NP otopathogens isolated at healthy visits occurring within one month before AOM

In order to determine whether the presence of bacteria in NP samples obtained at healthy visits shortly prior to onset of AOM might be predictive of the etiology of AOM, we compared data from 81 AOM cases where NP samples were taken within one month before an AOM but not at onset of AOM. 31 (42%) of healthy NP colonized children had the same pathogen in the MEF during their AOM visit as they had at the healthy visit. We analyzed the correlation between concordance of MEF culture results and NP cultures taken at healthy visits 4, 3, 2, and 1 week prior to AOM onset. NP cultures taken 1 week prior to onset of AOM were more frequently concordant with MEF cultures compared to NP cultures taken 2 weeks prior to onset of AOM. As the time interval between NP culture sampling and onset of AOM lengthened the concordance became significantly lower (p < 0.05).

### Correlation of MEF otopathogens with the NP at onset of AOM

To determine whether NP cultures correlated with MEF cultures at onset of AOM, 519 AOM cases out of 530 cases were compared. In 165 (31.8%) cases there was an exact match of MEF and NP isolate. In 359 (69.2%) cases at least one otopathogen in the NP sample matched the MEF culture result. In 160 (30.8%) cases no match was observed between the NP and MEF.

The otopathogens in the MEF where exact matches were observed in the NP (N = 165) is shown in Table 2. The best correlation was observed for NT*Hi* (~37%). In 194 (37.3%) children there was partial agreement between the NP and MEF with 1 organism from the MEF found among 2 or 3 other otopathogens isolated from the NP. The predictive value of NP cultures according to otopathogen(s) in MEF is shown in Table 3.

**Table 2:** **Distribution of otopathogens from children with AOM where same otopathogens were observed in the NP and MEF site (N = 165)**

Correlation of MEF otopathogens with their NP site			
Otopathogens	Number	Percentage calculated with exact match of otopathogens with MEF total (n = 165)	Percentage calculated with total AOM cases (n = 530)
*S. pneumoniae*	37	22.4	6.98
Nontypeable *H. influenzae*	61	37.0	11.5
*M. catarrhalis*	29	17.6	5.5
NT*Hi* + *S. pneumoniae*	9	5.5	1.7
*M. catarrhalis* + *S. pneumoniae*	19	11.5	3.6
NT*Hi* + *M. catarrhalis*	4	2.4	0.8
NT*Hi* + *M. catarrhalis* + *S. pneumoniae*	1	0.6	0.2
Other bacteria	5	3.0	0.9

Note: In 354 (68.2%) AOM cases the MEF etiology didn’t match exactly with the NP site of the children.

**Table 3:** **Predictive values of bacteriologic results from NP and MEF according to the presence or absence of the main otopathogens in AOM**

Otopathogens detected	Prevalence (NW/MEF)				PPV	NVP	Sensitivity	Specificity
	+/+	+/-	-/+	-/-	95% Confidence intervals			
*S. pneumoniae*	127	133	19	251	48.85	92.96	86.99	65.36
					4.62 to 55.1	89.23 to 95.71	80.42 to 91.98	60.37 to 70.12
NT*Hi*	170	83	17	260	67.19	93.86	90.91	75.80
					61.03 to 72.94	90.35 to 96.38	85.84 to 94.61	70.91 to 80.24
*M. catarrhalis*	93	159	10	268	36.9	96.4	90.29	62.76
					30.93 to 43.19	93.48 to 98.26	82.87 to 95.24	57.99 to 67.36

Note: PPV: Positive predictive value. NPV: Negative predictive value.

### Comparison of otopathogens with age

**Figure 1:**
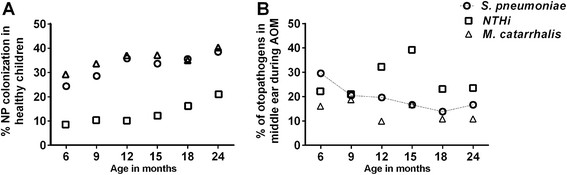
**Distribution of** ***S. pneumoniae*** **, NT** ***Hi*** **and** ***M. catarrhalis*** **bacteria at 6, 9, 12, 15, 18 and 24 months of age during healthy colonization and their presence in MEF during AOM.** The healthy colonization of each bacteria increased significantly with age but no significant difference was observed with age during AOM. A negative trend with age was observed for *S. pneumoniae* in MEF during AOM shown by dotted line in the figure.

The presence of *Spn*, NT*Hi* and *M*cat in the NP (Figure 1A) and their presence in MEF during AOM (Figure 1B) were compared according to the age of the child. During healthy visits NP colonization by potential otopathogens significantly increased with age (*p* <0.05, with correlation r^2^ = 0.7880 for *Spn*, 0.931 for NT*Hi* and 0.729 for *Mcat*). During AOM, the otopathogens in the MEF between 6-24 months of age did not show any age specific distribution. A negative trend with age was observed for *Spn* (p = 0.06 and r^2^ = 0.617) but NT*Hi* and *Mcat* were uniformly distributed between 6-24 months of age.

### Antibiotic susceptibility of NP isolates at AOM compared to NP isolates at healthy visits

Recovery of *Spn* in the NP during healthy visits was not significantly different from recovery the NP during AOM visits. However, among NP cultures at healthy visits 187 (28.5%) of 656 *Spn* isolates were oxacillin resistant compared to 98 (38.3%) of 256 of NP cultures at onset of AOM (p = 0.004). The number of *Spn* detected during NP colonization with a MIC ≥2 μg/ml to penicillin at healthy visits was significantly lower 34 (6.7%) of 509 isolates compared to 205 *Spn* isolates at AOM visits (12.7%). Recovery of NT*Hi* in the NP during healthy visits was significantly lower from the NP at onset of AOM (p < 0.0001). During healthy visits 57 (22.5%) of the 253 NT*Hi* isolates were β-lactamase positive compared to 72(32.3%) at onset of AOM (*p* = 0.016). The frequency of recovery of *Mcat* in the NP during healthy visits was not significantly different from recovery in the NP during AOM visits and in almost all visits *Mcat* was β-lactamase positive.

### Antibiotic susceptibility of middle ear fluid isolates compared to NP isolates at healthy visits

Oxacillin resistance of *Spn* in the MEF from AOM visits was not significantly different from *Spn* isolates at healthy visits. 42 (27.6%) of 152 *Spn* isolates from MEF at AOM visits were oxacillin resistant compared to 187 (28.5%) of 656 *Spn* isolates from NP at healthy visits. Comparison of *Spn* with MIC of ≥2 μg/ml to penicillin in MEF isolates and NP isolates at healthy visits showed 17.5% of 103 MEF isolates of *Spn* were penicillin-resistant compared to 6.7% of 509 NP isolates at healthy visits, which was significantly different (p = 0.001). 73 (37.2%) of 196 NT*Hi* isolates from MEF at onset of AOM were β-lactamase positive compared to 57 (22.5%) of the 253 NT*Hi* NP isolates during healthy visits (*p* = 0.001).

### Antibiotic susceptibility of middle ear fluid isolates compared to NP isolates at onset of AOM

42 (27.6%) of 152 *Spn* isolates from MEF were oxacillin resistant. 98 (38.3%) of 256 isolates of *Spn* from the NP at onset of AOM were oxacillin resistant, significantly lower comparing MEF isolates with NP cultures at onset of AOM (p < 0.001). Comparison of *Spn* with MIC of ≥2 μg/ml to penicillin in MEF and NP isolates showed 17.5% of 103 MEF isolates of *Spn* were penicillin-resistant compared to 12.7% of NP isolates at onset of AOM, which was not significantly different (p = 0.612). 73 (37.2%) of 196 NT*Hi* were β-lactamase positive. The proportion of β-lactamase positive in NT*Hi* MEF cultures was not significantly different from NP cultures collected at onset of AOM (p = 0.287). To calculate whether antibiotic resistance of MEF pathogens can be predicted from NP isolates obtained at onset of AOM, we compared oxacillin resistance in 127 paired NP and MEF *Spn* isolates and found the PPV to be very high at 95.3%. Similarly comparison of β-lactamase activity of 170 paired isolates of *NTHi* showed the predictive value at 96%.

## Discussion

Historically during the early 1970s to early 1990s, the relative proportion of otopathogens in MEF during AOM was 40% for *S. pneumoniae*, 25% for *H. influenzae* and 12% for *M. cattarrhalis* [20]. After the introduction of PCV-7 vaccine in the early 2000’s, changes in the frequency and distribution of AOM pathogens occurred [15],[17],[21],[22]. We sought to determine if NP cultures could be used to predict MEF cultures in the post PCV era. In this study from June 2006-August 2013, the relative proportion of otopathogens in MEF during AOM was 28.7% *Spn*, 37% *NTHi* and 19.6% *Mcat*. Thus our group and others have shown the introduction of PCV-7 and more recently PCV-13 has reduced the contribution of *Spn* causing AOM [15],[17],[22]-[24].

Can NP samples taken during healthy visits serve as a surrogate for MEF cultures? The results from the present study show that predicting the etiology of AOM in MEF cultures by analyzing NP cultures at healthy visits is poor and cannot be recommended as a substitute strategy for collection of MEF.

Are NP cultures taken at onset of AOM concordant with MEF cultures? NP cultures collected at onset of AOM to predict MEF culture results are somewhat better than NP cultures collected at healthy visits. The PPV for NP cultures at onset of AOM compared to MEF was 48.8% for *Spn,* 70.6% for NT*Hi* and 36.9% for *Mcat*. NPV values for all the pathogens were high.

Can antibiotic susceptibility of otopathogens collected from the MEF be predicted by otopathogen isolates obtained from the NP at healthy or at onset of AOM visits? Our data show poor correlation in predicting the antibiotic resistance of microorganisms in the MEF compared to NP samples taken during healthy visits. In comparison we found antibiotic susceptibility of otopathogens collected from the MEF can be predicted by otopathogen isolates obtained from the NP at onset of AOM visits.

We postulated and found that the correlation of NP otopathogens isolated at healthy visits occurring within one month before AOM would be stronger than cultures taken at times further distant from onset of AOM. The results are consistent with prior work showing that typical pathogenesis of AOM involves recent (generally < 2 weeks) acquisition of a potential otopathogen progressing to cause the infection [25],[26].

We observed that *Spn and NTHi* NP colonization increased significantly between 6 and 30-36 months of age, but as children got older the relationship between detection of a potential otopathogen in the NP with detection in MEF got weaker. Our results are in agreement with Syrjanen *et al* [27] who also found a high prevalence of NP colonization but low frequency of *Spn* as an etiology of AOM with age, especially after children became >18 months of age. However, neither our results nor those of Syrjanen *et al* [27] allow confidence to use NP cultures as surrogates of MEF cultures in children below 2 years of age. Our results are consistent and also support a Finnish study by Kilpi *et al* [28] where they showed that the incidence of *Spn* AOM peaked at 12 months of age, whereas the incidence of *NTHi* AOM peaked later at 20 months of age.

Our study has potential limitations. Our results were obtained from a single community and mostly from a single private pediatric practice. However, we have compared our MEF culture results in the past to those of Bardstown KT [29],[30], Pittsburgh PA [31] and Fairfax VA [32],[33] where tympanocentesis has been performed and found results to be similar [22]. Also, while participating in multicenter trials of new antimicrobial agents and pneumococcal conjugate vaccines during the 1990s and 2000s, our center had MEF culture results similar to multiple other U.S. centers but not to centers outside the U.S. where tympanocentesis was performed [34]-[37].

## Conclusions

In conclusion, the overall results bring into question the epidemiologic value of NP cultures to predict otopathogen mix or anticipated antibiotic susceptibility patterns among otopathogens. The results published here extend and confirm those of another recent paper from our group [17] in showing that NP isolation of otopathogens at onset of AOM better reflect, albeit incompletely, likely MEF isolates compared with NP isolates at times of health. We will continue to collect MEF at our otitis media research center for the coming years and collect NP cultures in order to provide results to the health care community for review and consideration in recommendations for AOM management.

## Authors’ original submitted files for images

Below are the links to the authors’ original submitted files for images. Authors’ original file for figure 1
