# Supplementary figures and images for: Correlation of nasopharyngeal cultures prior to and at onset of acute otitis media with middle ear fluid cultures
Source: BMC Infect Dis. 2014 Dec 5;14:640. doi: 10.1186/s12879-014-0640-y (PMC4264249; doi:10.1186/s12879-014-0640-y)

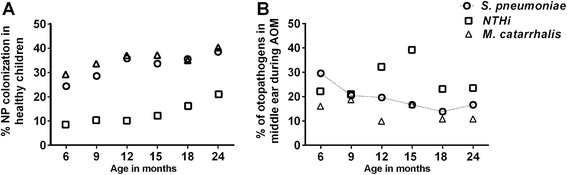

Supplement: Supplementary file 1 — Authors’ original file for figure 1 [file 12879_2014_640_MOESM1_ESM.gif]
